# Criteria used to categorise proteins as allergens for inclusion in allergenonline.org: a curated database for risk assessment

**DOI:** 10.1186/2045-7022-4-S2-P12

**Published:** 2014-03-17

**Authors:** Richard Goodman, Ronald van Ree, Stefan Vieths, Fatima Ferreira, Motohiro Ebisawa, Hugh A Sampson, Joseph Baumert, John Wise, Steve L Taylor

**Affiliations:** 1 University of Nebraska-Lincoln, Food Allergy Research and Resource Program, Lincoln, USA; 2 Academic Medical Center, Dept. of Experimental Immunology, Amsterdam, Netherlands; 3 Paul-Ehrilich-Institut, Div. of Allergology, Langen, Germany; 4 University of Salzburg, Christian Doppler Laboratory for Allergy Diagn, Salzburg, Austria; 5 Sagamihara National Hospital, Clinical Research Center for Allergy and Rheumatol, Sagamihara, Japan; 6 Icahn School of Medicine at Mount Sinai, Division of Allergy, New York, USA; 7 University of Nebraska—Lincoln, Food Allergy Research and Resource Program, Lincoln, USA

## Background

New proteins introduced in foods through genetic engineering or processing are often evaluated for potential risks of eliciting food allergy (Codex, 2003). The primary risk is the transfer of an allergen or a protein nearly identical to an allergen and capable of causing IgE mediated cross-reactions into a new food source. The AllergenOnline.org database was developed to provide a curated set of allergenic sequences for bioinformatics comparisons to identify proteins that should be tested further (e.g. serum IgE binding). Curation is necessary as the scientific literature and general sequence databases include thousands of proteins labeled as allergens that lack proof of allergenic activity.

## Methods

In 2006 we developed guidelines for reviewing and classifying candidate proteins as "allergens", "putative allergens" and those with "insufficient evidence" of causing IgE mediated allergic reactions in humans. Airway, contact, venom, salivary and food allergens are included. Criteria were developed for judging allergic subjects; allergen sources; protein characteristics, sequences; allergenic activity and IgE binding. Candidate allergens and peer reviewed publications are identified from the NCBI Protein and PubMed databases using keywords for review by our panel. Data, evaluations and decisions are stored in an achieved data management system during an annual update process. Access and FASTA searches are free, anonymous and unrecorded.

## Results

We evaluate: descriptions of allergic and control subjects (numbers and ages; types of reactions); symptom onset; route of exposure (contact, ingestion, inhalation or injection); diagnostic methods (history, skin prick test or other challenge, specific IgE, basophil activation). We consider allergen source information (taxonomy, tissue source, maturity or processing); evidence the protein is present in the test material; protein characterization and source (purified vs. extract, native vs. recombinant); sequence and methods (cDNA or protein sequence); molecular mass and glycosylation status. Specific IgE measurements should include standards, controls, details of critical reagents and scoring criteria. Tests should purified proteins as well as extracts to demonstrate relevance. Four examples are presented.

## Conclusions

Data quality and evidence of the importance of individual allergens varies markedly while the rate of publication of poorly described allergens and sequences is rising rapid.

